# Estimating the influence of the network topology on the agility of food supply chains

**DOI:** 10.1371/journal.pone.0218958

**Published:** 2019-07-10

**Authors:** Juan M. Hernández, Carmen Pedroza-Gutiérrez

**Affiliations:** 1 Department of Quantitative Methods in Economics and Management, Universidad de Las Palmas de Gran Canaria, Las Palmas, Spain; 2 Institute of Tourism and Sustainable Economic Development (TIDES), Universidad de Las Palmas de Gran Canaria, Las Palmas, Spain; 3 Escuela Nacional de Estudios Superiores-Mérida and Unidad Académica de Estudios Regionales, Coordinación de Humanidades, Universidad Nacional Autónoma de México, Jiquilpan, Michoacán, México; Shandong University of Science and Technology, CHINA

## Abstract

Several studies have shown that the performance of a supply chain is heavily influenced by the pattern of relationships among firms. This paper analyzes the structure of relationships (network topology) that leads to the highest agility of a food supply chain when sudden demand changes occur. To do this, a simulation model that represents a supply chain and specific rules to allocate orders is built. The supply chain in the model follows the specific characteristics of trade in the primary sector. The model is fitted to the conditions of a real seafood supply chain in Mexico. Agility is measured through the effect on the order fulfillment of a sudden demand shock and the recovery time of this rate to previous values. The simulation results show that the most suitable structure depends on how product is distributed among suppliers. If product is evenly shared, supply chains with homogeneous topologies are more agile than supply chains with heterogeneous topologies, but the result is the opposite if product is unevenly shared among suppliers. Other previous recommendations, such as having multiple suppliers and horizontal links, are confirmed by the simulations. These findings contribute to the general debate on which is the optimal topology for an agile supply chain.

## Introduction

Supply chain (SC) has been conceptualized as a network of agents (e.g., suppliers, manufacturers, distributors, retailers and consumers) who are interconnected throughout the transference of material or information [1]. This perspective departs from the dyadic (buyer-supplier) and triadic view which center on the particular relationships between categories [2,3] to a more extended focus where the object of analysis is the supply chain as a whole, represented by a collection of nodes (agents) and links (transactions among them).

Following the network perspective, a supply chain is also termed supply network [4,5]. A supply network is represented by a complex system of interdependence among multiple firms, each one with a number of links to specific partners. In this context, the performance of a supply network is not just the result of an additive aggregation of the individual firms’ decisions, but a coherent and autonomous global behavior emerges from the non-linear interaction among agents [6,7]. This behavior is dependent on the specific structure of the total relationships among agents in the supply network, what is called the network topology. Several models and empirical studies have been developed in the last two decades following the supply network perspective [8].

In this paper, we analyze the influence of the network topology on the agility of a supply chain. SC agility can be defined as the ability to respond quickly to sudden changes in supply and demand and has been recognized as one of the key qualities for top-performing supply chains [9]. Agility is usually analyzed from a focal agent’s view, assuming the position of a single company in the network and looking at the surrounding supply chain in relation to this company. Instead, we extend the focus and analyze agility of the network as a whole, not from the perspective of a single agent [7].

The supply networks studied in this paper follow the features of some food supply chains (FSCs), where the relationships among agents are usually more restrictive than in industrial SCs. In particular, the product and information in these FSCs need to pass through intermediaries and bypass seldom occurs. This paper identifies the conditions in which, in this kind of supply networks, some types of topologies present higher levels of agility than others. Specifically, the main contribution of the paper is that, contrary to what is commonly believed in the specialized literature, SCs with heterogeneous distribution of links (characterized by few agents with many relationships combined with many agents with few relationships) are not necessarily the most agile ones.

This paper also contributes methodologically by building a simulation model to estimate agility, where the SC admits a large number of agents and includes the complexity of interdependence among them. Specifically, the SC has three tiers (suppliers, wholesalers and retailers) and the pattern of relationships among agents in different tiers follows some probabilistic distributions. The theoretical SC is built through the realization of random network models in bipartite graphs [10]. The model parameters and probabilistic distributions are fixed to resemble the data from a real seafood SC in a region of Mexico. Several patterns of relationships between tiers are considered. The results show the differences in the SC adaptation to demand shocks when assuming alternatively heterogeneous and homogeneous distributions of links.

The paper is organized as follows. Next section presents a literature review. Section 3 explains the model to test agility in a specific probabilistic SC. Section 4 shows the empirical SC and the simulation results. Last section is devoted for discussion and conclusions.

## Literature review

### Supply chain agility

Agility emerged in the beginning of the 1990 as a solution for managing a dynamic and changing environment of the enterprise [11]. Several definitions of SC agility have been given throughout the last two decades, each one incorporating new aspects to an underlying original idea [12]. Each definition aims to identify the main attributes, capabilities and practices that comprise the agile enterprise/organization or in other words the supply chain practices that are described as agile enterprise [13].

Jackson and Johansson [14] divided agility capabilities into four dimensions: product-related change capabilities; change competency within operations; internal and external co-operation and; people, knowledge and creativity. Among these dimensions, our paper focuses mainly on the external co-operation, understood as the ability to cooperate with suppliers and customers. Therefore, we refer to the inter-enterprise level, or inter-organizational cooperation of the supply network [15]. In other words, to the total relationships among agents in the supply network who are interconnected through material or information transactions [1].

One of the main attributes related to agility is responsiveness, which is defined by Swafford, Ghosh, and Murthy [16] as ‘the supply chain’s capability to adapt or respond in a speedy manner to a changing marketplace environment’. These authors also posit that flexibility works as an antecedent of SC agility, being flexibility a competency and agility a capability. Moreover, flexibility includes several dimensions, such as the range of states a system can adopt and the ability to change in a costly and timely manner.

The concept of flexibility has been also recurrently included as an ingredient of agility in the broader context of SC resilience [17–19]. For example, Pettit, Fiksel, and Croxton [17] define flexibility as the ‘ability to quickly change inputs or the mode of receiving inputs’ (flexibility in sourcing) and the ‘ability to quickly change outputs or the mode of delivering outputs’ (flexibility in order fulfillment). According to Sheffi and Rice Jr [20], flexibility can be achieved by working with multiple suppliers and having multiple capabilities at each plant location. These strategies are related to the concept of redundancy, which refers to the existence of extra stock (high inventory levels) or having multiple suppliers.

The conceptualization given by Swafford, Ghosh, and Murthy [16] above refers to the operational aspects of agility. On the contrary, Christopher, Lowson, and Peck [21] emphasize the informative aspects of the concept and define an agile SC as market sensitive (closely connected to consumer’s trends), with virtual integration through shared information among all players in the SC, network based or with flexible arrangements with suppliers, and process aligned, where all partners are highly coordinated. The role of cooperation, coordination and communication as enablers of SC agility has also been theoretically founded and empirically validated by [12].

Thus, collaboration among the different entities in the SC positively influences on agility. Collaboration can be vertical, if produced among agents in different tiers of the supply chain, or horizontal, if produced among entities in the same tier. Horizontal relationships have been observed in food processing companies and are justified by the incentive of receiving back the help in times of crisis [22]. The collaboration between competitors implies the transaction of information and material between partners when needed, what enhances flexibility and consequently agility. Moreover, Yusuf et al. [13] found that one of the factors that characterize the agile pattern in SC is a high degree of cooperation with competitors. These are relationship-oriented aspects of agility associated to environments emphasizing flexibility.

Both facets of agility (operational and informative) were unified by Li et al. [23], which identify two components in an agile SC: (a) Alertness to changes; and (b) Response capability. The former refers to the ability to anticipate threats and opportunities in the supply chain or marketplace and the latter refers to the capability to use resources in responding to changes. The mode to set up agility can be proactive (anticipating market changes) or reactive (responding to market changes). Gligor, Holcomb, and Stank [24] extend the dimensions of agility, but maintaining the two main aspects, which rename as cognitive and physical categories. The cognitive dimensions include alertness as well, but add other two: The ability to access to information (accessibility) and to make decisions (decisiveness). The physical dimensions are flexibility and swiftness (also called speed and velocity by other authors).

### A topological metric for SC agility

Several instruments to measure SC agility have been proposed up to date by applying different methodologies: (a) Scale development process, where a sample of items founded on theory are generated and empirically validated by means of a survey conducted among firm managers [16,24,25]; (b) Fuzzy logic methods, which transform linguistic valuation of certain attributes into numerical objects, which are mathematically treated to obtain a global valuation of the SC agility [26,27]; (c) Graph theory tools, applying the permanent function on the matrix of interdependences of agility’s enablers and criteria [28].

These methods are suitable for dealing with subjective and multidimensional concepts, such as SC agility. However, the metric obtained is based on expert knowledge of every specific SC, which makes it non-applicable for theoretical supply network models, such as the one analyzed in this paper. In these network models, an objective, precise and quantitative agility metric is necessary to compare the simulation results for the different numerical scenarios proposed.

In this paper, we follow the network perspective to analyze SC agility. Consequently, the metric used here takes into account the topology of the supply network. Generally speaking, a network is a set of vertices (nodes), edges (links) and the way (topology) both elements are connected. In the context of SCs, nodes are represented by firms, while edges represent relationships among those agents. These relationships can be formalized through the existence of some flow of material, information or contracts among the parts [5,29]. For simplicity, in this paper we assume the same network structure for the three types of relationships. The social network analysis [30] makes use of quantitative indicators to identify key nodes and the general characteristics of the network structure.

Up to date, few specific topological metrics for SC agility have been proposed. Some authors have used average supply path length (ASPL) embedded in a mix of metrics of resilience in a supply network [31–34]. Although not explicitly stated in the previous contributions, ASPL could serve as an initial metric for SC agility. Since every firm that manages a product includes some delays associated with receiving, processing and delivering, a low average supply path length means that the material passes through few hands, therefore reducing the total delay and increasing agility.

Other metric is the Supply Chain Index (SCI), proposed by Plagányi et al. [35]. This metric includes the flow throughout the SC in the definition. Low values of SCI indicate that the flow is diffused among multiple agents, while large values imply high concentration in few nodes. The SCI was tested with several simple examples of linear SCs. The authors find that the most favorable values of SCI in terms of agility depend on the form of adaptation cost of changes. In general, diffuse flows favor quick adaptation to changes if the cost of changing flows from one agent to another is not scale dependent.

However, as metrics for SC agility, the two indexes above suffer some relevant shortcomings. Specifically, they do not explicitly consider the effect of changes in demand or supply on the SC performance. Moreover, time evolution is not included either and consequently backorders and recovery time are disregarded. Transportation of material takes some time, which is essential for analyzing the response of the SC when a change occurs. Thus, lead-time or the total time to complete and distribute the product has been identified by a panel of experts as the major determinant of agility [36].

The percentage of fulfilled demand over the total demand, also called order fulfillment rate (OFR), can overcome part of the previous deficiencies. This metric has been traditionally used as a quantitative SC performance measure [29,37]. In a more recent empirical analysis conducted in the Volkswagen Group, Cabral, Grilo, and Cruz-Machado [38] have found that OFR, together with the “responsiveness to urgent deliveries”, are the most relevant criteria to get a system of rapid response and consequently an agile SC. Some other authors propose OFR as part of other resilience measures of SC [39,40]. More recently, in a similar vein to this paper, Ledwoch, Yasarcan, and Brintrup [41] proposes an agent-based simulation model and uses OFR as a performance metric of the supply network when supply disruptions occur.

In order to include the effect of changes in demand and the time factor, this paper makes use of OFR along a time span to measure SC agility. We assume exclusively a sudden shock in demand in a fixed time and observe OFR before and after the shock. The flow of material throughout the SC in every time step is determined by the difference between the current demand from the final customer, which is added to backorders from previous periods, and the distribution capacity of every firm. The immediate effect of the demand shock on OFR and the recovery time will be used as metrics for agility. The former (immediate effect) determines the ability to manage sudden shocks in demand, while the latter (recovery time) represents the velocity of adaptation to the shock.

Thus, these metrics serve as indicators of what is called ‘response capability’ by Li et al. [23] and ‘physical dimensions’ by Gligor, Holcomb, and Stank [24], both presented in the previous section. In addition to swiftness, flexibility in the SC can be measured by comparing the immediate effect on the OFR and recovery time of the different supply network structures when a sudden shock occurs. This will be done in the simulations below. However, other dimensions of SC agility are not included in these metrics. This is the case of the cognitive/informative dimensions of SC agility (e.g., alertness, decisiveness). Moreover, OFR quantifies reactive capacities of the SC, but not proactive nor alertness, in Li et al. [23] terms. Thus, the metrics are close to the operational level given in [16]. Nevertheless, the effect of collaboration on SC agility can be estimated by observing the changes on OFR and recovery time when increasing horizontal or vertical links among agents. Unlike to previous papers using simulation models of supply networks [41], we use these metrics to estimate the effect on agility of sudden shocks on demand, leaving aside shocks on supply.

### Characteristics of the food supply chain (FSC)

The food sector has its own particularities which make it a challenging domain from a supply chain management perspective. One of them is that the FSC links the agricultural, food processing and distribution sectors, and by managing these links it has to meet consumers’ demands on healthy and fresh products [42]. Producers must better and more efficiently respond to the increasing and changing demands from the markets.

Food supply networks have to deal with perishable products, unpredictable supply variations, stringent food safety and sustainability requirements [43], in addition to fresh food degradation which depends on the way food is processed, store and transport. In this sense, a major concern in the FSC is consumers’ satisfaction which is highly based on their judgment about food quality, making quality the most essential food product characteristic to consider throughout the supply chain [44]. An agile distribution is essential to preserve the freshness of food, avoid its degradation and maintain its quality.

The FSCs commonly include wholesale centers, located in large urban areas, which buy and distribute products through a variety of suppliers and retailers, and many of the traded goods come from small-scale producers from rural economies in developing countries. To be able to sell their products, these producers participate in a multi-tiered SC, where middlemen are key actors that connect producers with external markets. Middlemen act as facilitators of trade between producers and wholesalers. To give some examples, this market structure has been identified in many rural economies in agriculture and fishing, such as the potato market in Ethiopia [45], the rice in Philippines [46], or the fisheries Malaysia [47], Kenia [48] and Mexico [49].

The reasons why small-scale producers trade their products through middlemen/wholesalers and the influence that these intermediaries have in the FSC are from socioeconomic nature. Small-scale producers in these economies have low production volumes, limited trading skills and lack of preservation or transportation facilities. These factors do not help the small-scale producers to organize and have fluid horizontal relationships among them, which might allow them reaching local, national or international markets.

Middlemen are a social optimal choice to decrease transactions costs, specifically those related to search and matching costs [45,50,51]. Middlemen act as a time saving institution by shortening the negotiation time of sellers and buyers in a transaction [52]. Moreover, they are considered important providers of market information [45], match geographically dispersed buyers and sellers [51], have better information and in general have the ability to contract separately with upstream and downstream parties and achieve supply and delivery coordination, in sum they have higher expertise [50].

Thus, FSCs are structured in such a way that wholesalers are supplied by middlemen, which in turn are supplied by producers. Finally, retailers buy product to wholesalers. Both middlemen and wholesalers act as intermediaries, although sometimes middlemen who trade high amount of fish work as wholesaler as well. Thus, in this kind of SCs a restricted relationship between agents exists, where every agent is supplied by other agents in the precedent tier and supply to agents in the subsequent tier. We call this structure *restricted relationship*. Thus, the type of SCs analyzed here are different to other real industrial SCs [5], in which more flexible relationships among firms are allowed.

**Fig 1:**
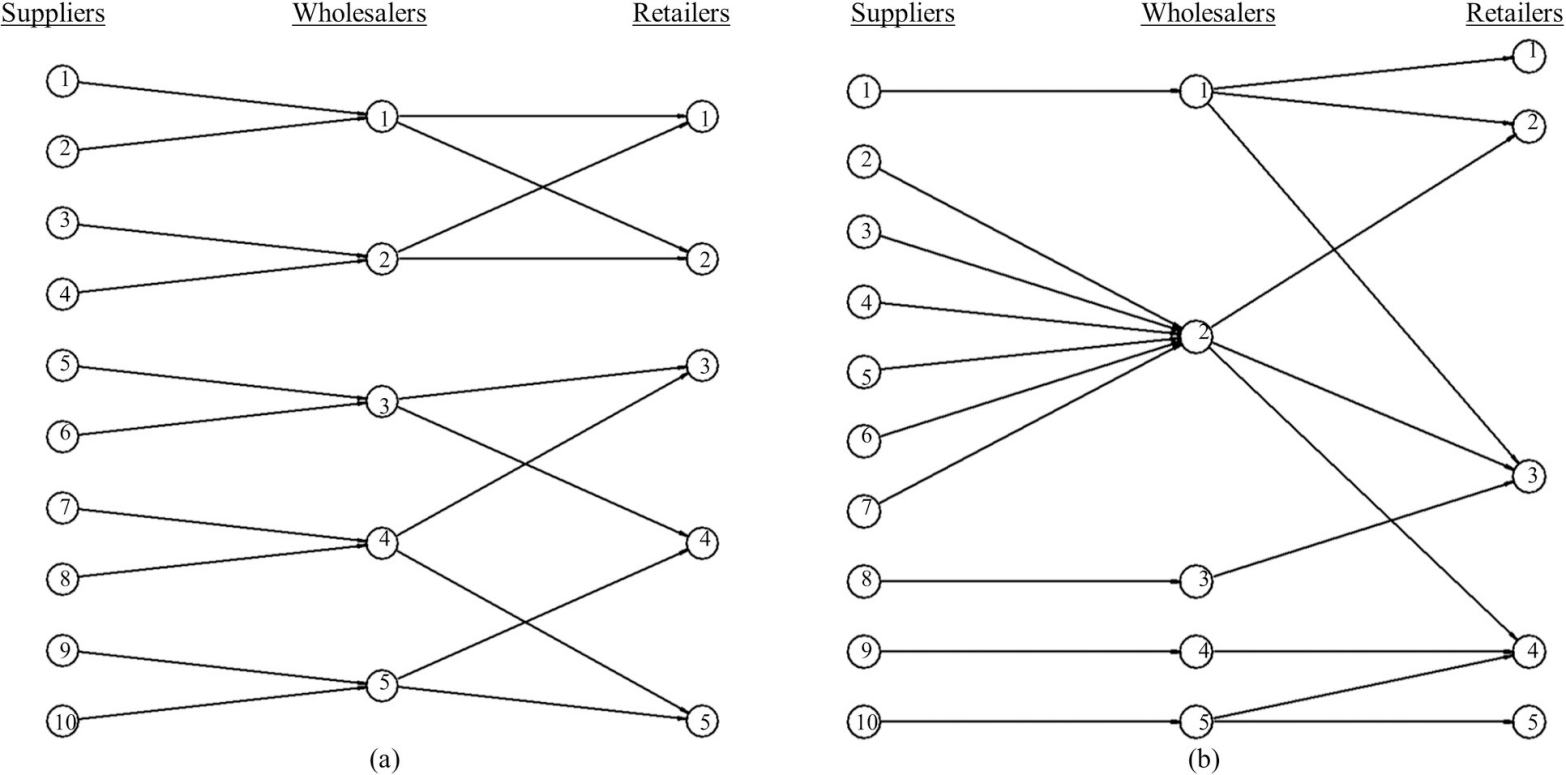
Two examples of a three-tiered supply network. The two supply networks follow different topologies in the wholesaler and retailer tier: (a) Homogeneous topology (regular degree distribution with $\bar{k}=2$); (b) Heterogeneous topology (power law degree distribution with $\bar{k}=2$).

Fig 1 illustrates two examples of supply network structures in a three-tiered SC with restricted relationship. In this Figure, the role of intermediaries is adopted exclusively by wholesalers, who buy product to suppliers and sell it to retailers. Fig 1A and Fig 1B show different patterns of links among firms in the supply network. The number of links from a node is called node degree and the degree distribution among nodes determines a topological structure. Fig 1A shows a realization of a regular degree distribution of links between supplier-wholesaler and wholesaler-retailer. This homogeneous distribution assumes a constant degree ($\bar{k}$) for any firm in the SC. On the contrary, Fig 1B shows a realization of a heterogeneous distribution of links, in this case the power law degree distribution. The network topology with heterogeneous distribution of links is characterized by the existence of few but significant amount of highly connected nodes together with low connected ones.

Empirical analyses reveal that some real SCs resemble the heterogeneous topology. This is the case for some automotive SCs, which show a highly centralized network structure [5], and the supplier-customer network in the Indian auto-component industry [53]. Recently, Brintrup, Wang, and Tiwari [54] have found that the Airbus supply network approximates a heterogeneous topology. Specifically, the degree distribution seems to follow a power law and consequently the topology would be scale-free, but the data size in that paper is too small to be conclusive. Although several theoretical analyses support the idea that efficient SCs present scale-free topologies [55], empirical evidences with large enough amount of data do not show that this topology is presented in real SCs [8]. For example, other studies with automotive networks find that the degree distribution is exponential [34] and log-normal [56]. Both distributions generate heterogeneous topologies, although not scale-free.

However, in the case of SCs with restricted relationships, homogeneous distribution of links may favor agility more than heterogeneous distributions. For example, in the case of Fig 1 and if all suppliers distribute identical quantity of product, when a sudden change in demand occurs, some wholesalers in Fig 1B may be overloaded of demand (e.g. wholesaler 1) while other bigger sellers can fulfill their orders making use of part of their capacity (e.g. wholesaler 2). Therefore, the total orders in the SC would not be fulfilled quickly although it had enough goods from the suppliers. This unbalanced response to demand is avoided with a homogeneous distribution of links among firms, such as the one illustrated in Fig 1A. In this case, the gap between the number of buyers and sellers in every firm is null, so the total unfulfilled orders could be reduced.

From these arguments, we state the first hypothesis of this paper:

H1: In case of similar quantity of product distributed by suppliers and absence of horizontal relationships, when a sudden shock in demand occurs, agility in SCs with restricted relationships and homogeneous-degree topologies is higher than in SCs with restricted relationships and heterogeneous-degree topologies.

In SCs with restricted relationships, *N* tiers and non-horizontal relationships, the path length from any supplier connected to the final demand is constant and equal to *N*−1, the number of links from the initial supplier to the final customer. If horizontal relationships exist, firms can buy and sell to other firms in the same tier when needed. Thus, other new alternative paths for the flow of material from the first supplier to the final customer are included, favoring a quick response when sudden increases in demand occur. Additionally, a larger number of vertical interrelationships among firms in different tiers also enhances agility, by increasing the range of states in the system [16].

Then, we posit the second hypothesis of this paper:

H2: When a sudden shock of demand occurs, the larger the number of horizontal and vertical relationships among firms, the more agile a SC with restricted relationship is.

## A model to test supply chain agility

In this section, we present a stylized network-based SC model, starting from which the response to demand shocks will be estimated. The model assumes a large number of firms interacting in three echelons. Additionally, the pattern of interrelationships among firms in subsequent echelons is probabilistic and follows some predetermined degree distribution. The SC agility obtained with different degree distributions will be then compared.

Since the model includes uncertain factors and is designed for a high-scale system, simulation is the selected methodology to obtain results. Simulation has been commonly used to analyze the SC performance in several contexts, such as when decentralized informational structure is implemented in the system [39] and when trust between agents determines the order and shipment allocation rules [57]. Other SC simulation models have been previously utilized for analyzing agility as well. For example, Helo [58] builds a linear SC to explain how agility can be implemented by extending capacity instead of augmenting inventory.

**Fig 2:**
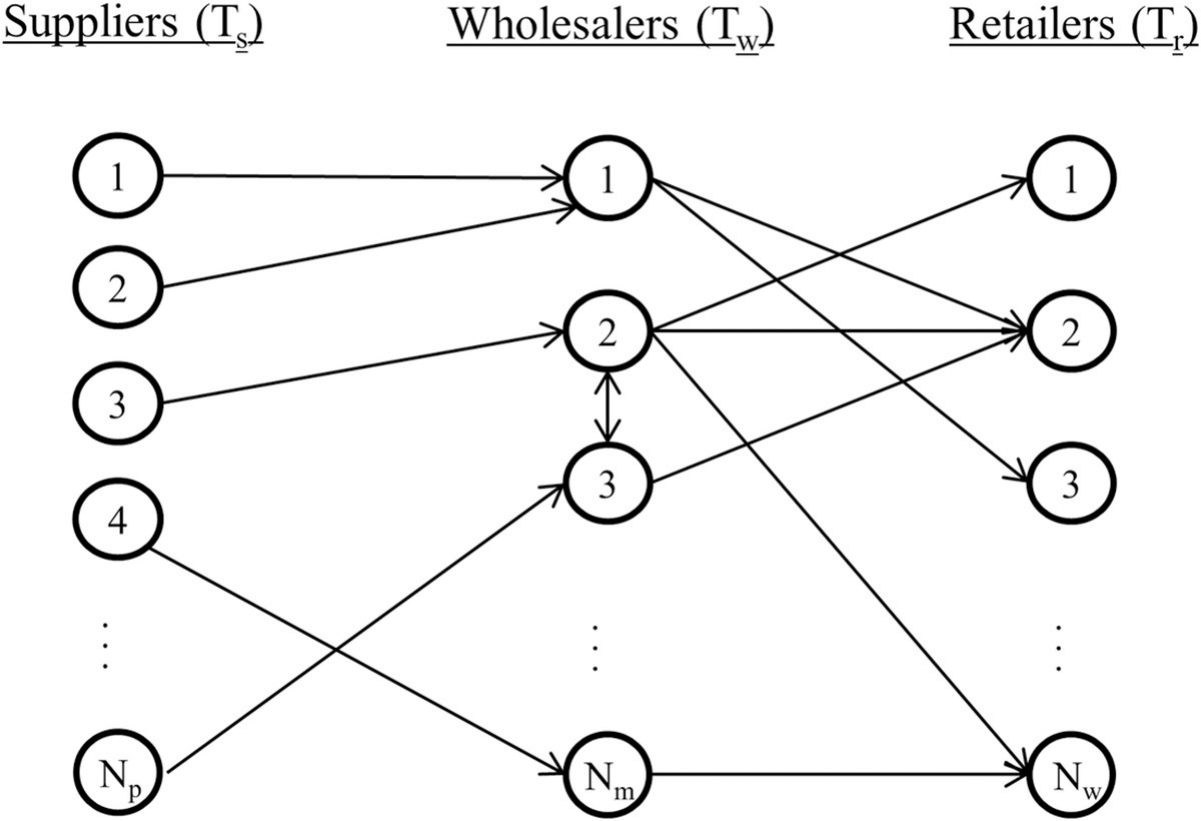
Representation of the supply chain random network. The SC includes three tiers: Suppliers (*T*_*s*_), Wholesalers (*T*_*w*_) and Retailers (*T*_*r*_). *N*_*s*_,*N*_*w*_ and *N*_*r*_ represent the number of suppliers, wholesalers and retailers, respectively. Arrows indicate flow and direction of material between firms. The bidirectional arrow indicates horizontal relationships.

Fig 2 shows the general representation of the model proposed, called supply chain random network (SCRN). According to a topological viewpoint [59,60], a supply chain is a directed random graph *G*(*V*,*E*), where *V* indicates the vector of tiers and *E* the set of links among nodes (firms) in the graph. In general, a supply chain can include multiple tiers, but as said above we limit to three in this model. So,

$$V=(T_{s},T_{w},T_{r}),$$

where *T*_*h*_ = {*h*/*h* = 1,2,…,*N*_*h*_}, with h = {*s*,*w*,*r*}, are the sets of nodes in the tier corresponding to supplier, wholesaler and retailer, respectively. These sets do not include any node in common and the number of nodes in every tier is not necessarily the same. We denote *α*:1:*β*, with *α*, *β* positive scalars, the ratio of the number of nodes between subsequent tiers (e.g., if *N*_*w*_ = 100, notation 3:1:5 indicates that the number of suppliers, wholesalers and retailers are 300, 100 and 500, respectively).

The links indicate the existence of a buyer-seller relationship between two firms, so some material (product) is transported between them when necessary and available. By assumption, links only connect nodes between subsequent tiers *T*_*s*_→*T*_*w*_→*T*_*r*_, so a direct nexus *T*_*s*_→*T*_*r*_ is not considered. Therefore, *E* includes pairs (*s*,*w*) or (*w*,*r*) if node *s* distributes product to *w*, and node *w* distributes product to *r*, with *s*, *w*, *r* in *T*_*s*_,*T*_*w*_,*T*_*r*_, respectively. The horizontal relationships are bi-directional, so the two linked firms in *T*_*w*_ can send product to each other in order to satisfy their corresponding demands. Thus, two firms linked with horizontal relationships function as one. By assumption, only one type of product is traded.

The SC topology is a combination of two bipartite random graphs [61]. They are the supplier/wholesaler and wholesaler/retailer random graphs. Every supplier *s* has $k_{s}^{out}$ links to the wholesalers and every retailer *r* has $k_{r}^{in}$ links from the wholesalers. Every wholesaler *w* has $k_{w}^{in}$ links from the supplier and $k_{w}^{out}$ links to the retailers. We assume that the degree distribution among nodes in every tier (two in-degrees and two out-degrees) follows some prescribed probability distributions. A sample of the random graph is obtained by following the same procedure used for the configuration model [10]: First, we generate the in- and out-degree of every node by simulating the specific distribution and every node is assigned a number of ‘stubs’ equal to its degree; Second, we randomly connect two stubs belonging to subsequent tiers, creating links among the nodes. To assure consistency (the sums of the degrees in subsequent tiers must be identical), the mean of the distribution times the number of vertices in subsequent tiers must be identical. Nevertheless, the specific sample may be inconsistent yet. In this case, one node from each one of the two tiers is dropped and their degrees are redrawn until the sample is consistent.

Every node in *T*_*s*_ and *T*_*w*_ includes a weight (capacity), which indicates the maximum volume of product that the node can distribute in a certain unit of time. We assume that the supplier capacity follows a probability distribution *c*_*s*_ with mean capacity $\bar{c_{s}}$. Every supplier distributes his/her entire product among the wholesalers he/she has relations with and wholesalers receive identical proportion of product from the supplier. Then, the product that wholesaler *w* receives from supplier *s* is $c_{w}^{s}=1/k_{s}^{out}∙c_{s}$. The wholesaler’s capacity *c*_*w*_ is the total amount of product he/she receives. In the example shown in Fig 2, wholesaler 1’s capacity is *c*_1_+*c*_2_, while wholesaler *N*_*w*_’s capacity is *c*_4_. Thus, the production is fully transported from suppliers to wholesalers and the average capacity of the second tier is $\bar{c_{s}}N_{s}$. For simplicity, the capacity of every supplier is assumed constant throughout time.

The model includes a dynamic evolution of the demand. The total demand in the system at every time step is *D*(*t*). Every retailer orders a fixed proportional part of the total demand according to the number of wholesalers he/she has relation with (${\bar{D}}_{r}(t)=k_{r}^{in}/N_{r}∙D(t)$). In other words, the more the number of links with wholesalers, the more the demand from a retailer is. The total demand from retailer *r* at time *t* is the sum of the fixed demand and backorders ($D_{r}(t)=\bar{D_{r}}(t)+e_{r}(t-1)$). The ratio $OFR(t)=1-\sum_{r=1}^{N_{r}}e_{r}(t)/\sum_{r=1}^{N_{r}}D_{r}(t)$ gives the percentage of fulfilled orders at time *t*.

**Fig 3:**
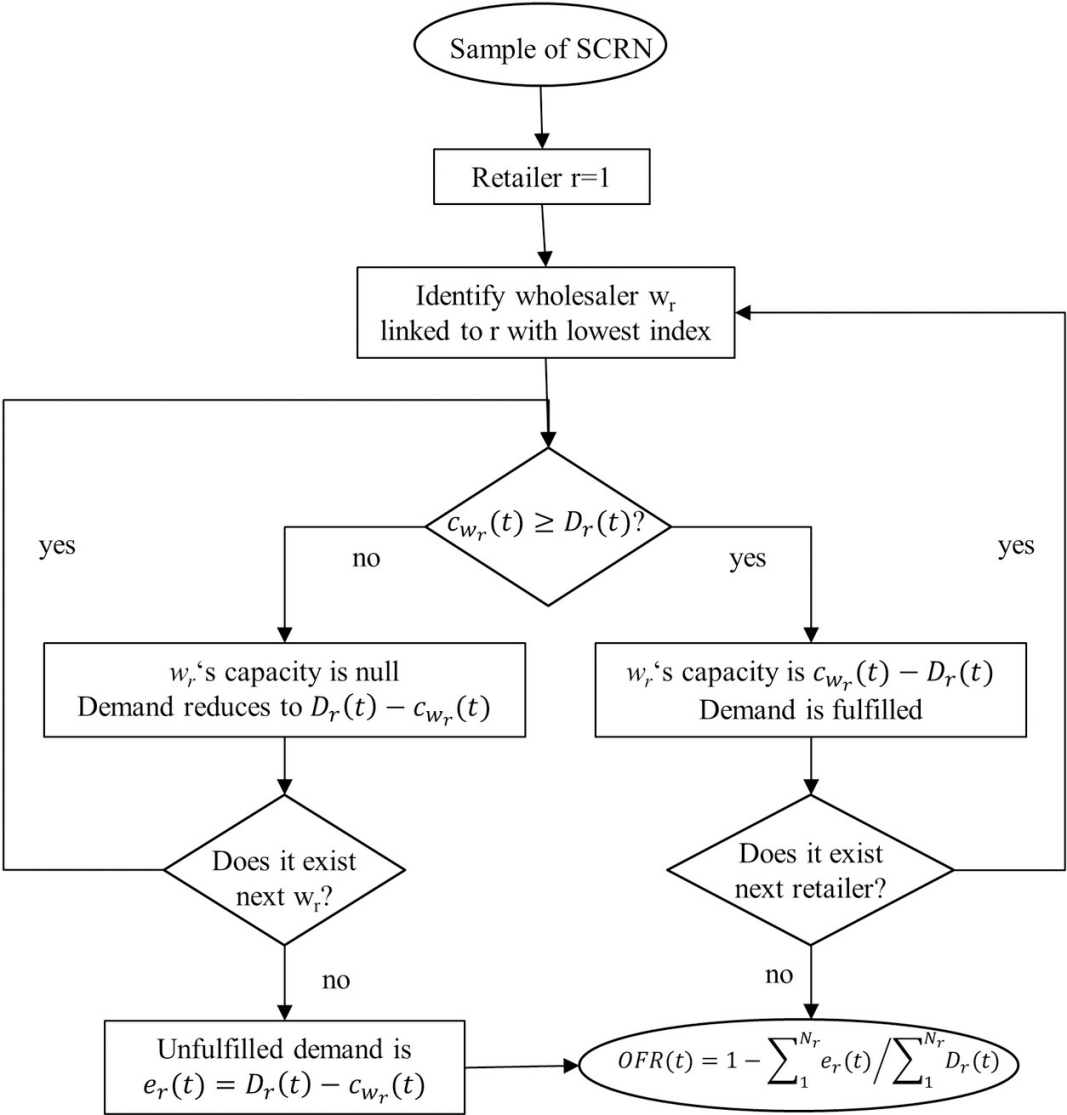
Flow chart. It illustrates the order allocation rule in the SCRN at every time step.

The order allocation rule in the SCRN at every time step *t* is represented in Fig 3. The supply allocation rule assumes a kind of hierarchy among retailers and wholesalers: Any retailer *r* has preference to be satisfied over all *r*′, such that *r*′>*r* and any wholesaler must satisfy all the demand from a particular retailer before attending the next retailer in the hierarchy; Likewise, any wholesaler *w* has preference to satisfy *r*’s order over all *w*′, such that *w*′>*w*.

The simulation model does not include delivery times and inventory levels in every firm. Thus, the excess capacity of every wholesaler is not accumulated in the next period. These restrictions fit quite well to the case of FSCs, where the perishability of the fresh product makes necessary short delivery times and impedes to keep it in stock for a long time.

For simplicity, we only assume demand changes to test the SC agility. These changes are in the expression of the total demand from retailers:

$$D(t)=\left\{ \begin{array}{c} \theta \bar{c_{s}}N_{s},\;t\neq t^{*},\\ \bar{c_{s}}N_{s},\;t=t^{*}, \end{array}\right.$$ (1)

where 0≤*θ*<1. So, the average capacity of wholesalers exceeds the demand at any time *t* excepting t*. At t*, a shock is produced and demand equalizes to the wholesalers’ capacity. The model provides *OFR*(*t*) along discrete time steps *t* = 0,1,…,*t*_*F*_, with t*<*t*_*F*_. Agility is measured through the percentage gap *ΔOFR*(t*) = (*OFR*(t*)−*OFR*(t*−1))/*OFR*(t*−1) and the recovery time (τ) to 95% of pre-shock values. In other words, the recovery time is the number of time steps after t* until the OFR firstly achieves at least 95% of its value in t*−1.

The model resembles the real distribution of product in FSCs, although with some restrictions. One of them is the hierarchy among retailers and wholesalers. Non-mechanistic factors influencing the selection of suppliers, such as cost or quality, are not included. Any kind of strategic behavior of the agents are not considered either. Nevertheless, the restricted relationships among tiers observed in FSCs and the diversity of topological patterns are well captured by the model.

## Empirical data, simulation setup and results

### Empirical data

Previously to the simulation setup, an empirical analysis of a real seafood SC was conducted. The structure of interrelationships among agents in the real SC will be used to determine the probabilistic functions in the model. Thus, the simulation results will provide insights about the agility of the real SC.

The seafood SC around the Mercado del Mar (MM) in Guadalajara, Mexico, was chosen. This is the second largest fish marketplace in Mexico, with a daily volume of trade around 500–1000 tons. The product comes from the own state (Jalisco), other Mexican states and foreign countries. Wholesalers in the MM sell their product mainly to local markets, restaurants, fish shops and wholesalers from other marketplaces in Mexico.

The SC was reconstructed based on several interviews made to a sample of 10 wholesalers in the MM (from a total of 44). We limited the scope of the analysis to the freshwater fish trade market. Wholesalers were asked about their trade relationships among suppliers and buyers. Additionally, part of the suppliers and buyers were visited in order to verify the information provided by wholesalers. In sum, 11 processing plants from two different states (suppliers) and 11 market stalls in Guadalajara (buyers) were interviewed. Ethics of this research were evaluated and approved by the Social Sciences and Humanities Coordination at the National Autonomous University of Mexico (UNAM). More information about the data can be found in [62], where the role of reputation in the MM is analyzed.

To standardize the information, suppliers were aggregated according to their location and the buyers were aggregated according to their sector (small retailer marketplaces, supermarkets and restaurants) and location. Nevertheless, some wholesalers declared to have relationships with agents whose location was not specified. They were included in a new unknown isolated agent, which exclusively trades with this wholesaler. A total of 27 suppliers and 56 retailers were identified in the sample. Approximately 50% of the wholesalers declared to have horizontal relationships as well.

**Fig 4:**
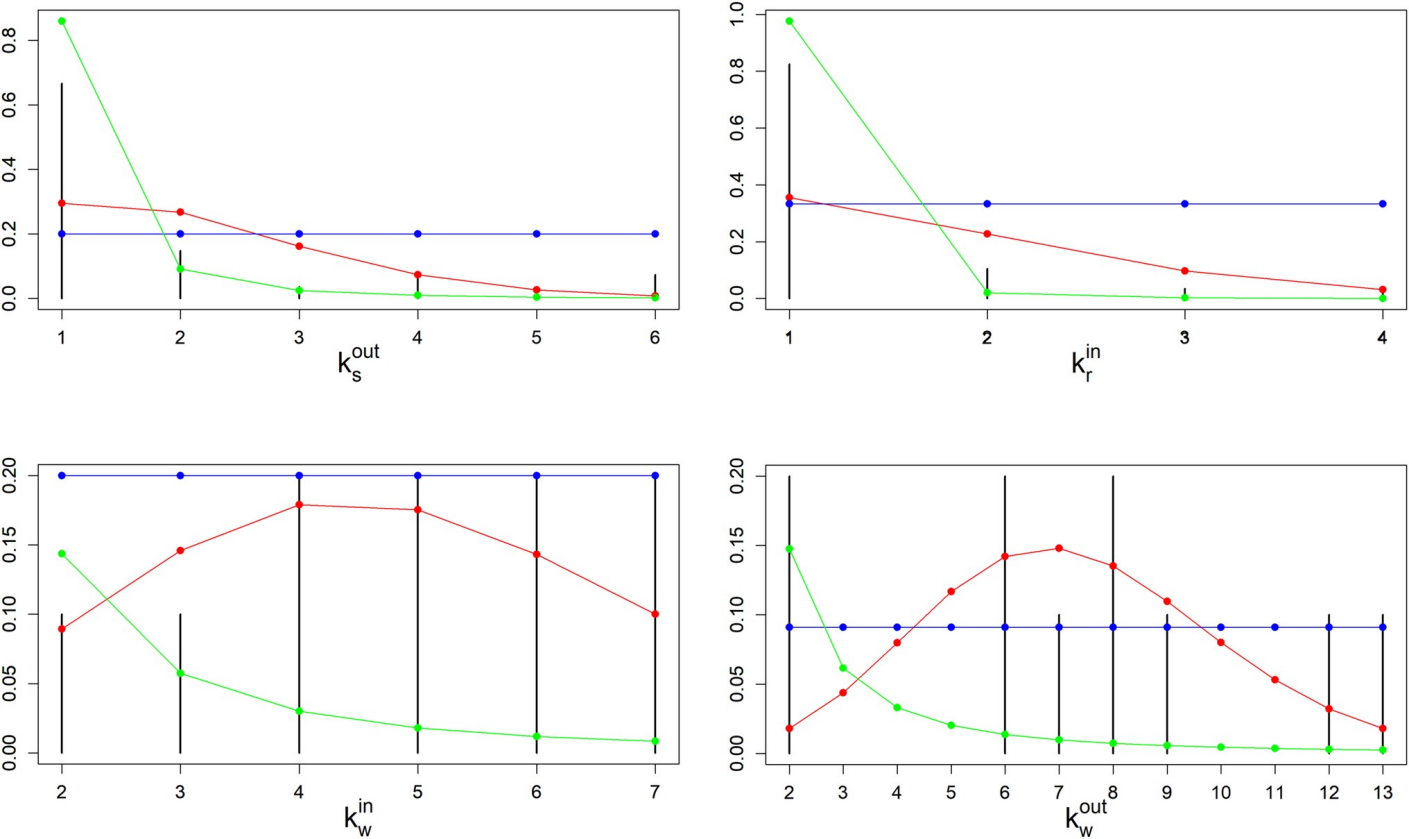
Empirical degree distributions of links in the SC of the Mercado del Mar, Guadalajara, Mexico. The variables are: $k_{s}^{out}$, out-degree distribution of suppliers; $k_{w}^{in}$, in-degree distribution of wholesalers; $k_{w}^{out}$, out-degree distribution of wholesalers; $k_{r}^{in}$, in-degree distribution of retailers. Three hypothetical degree distributions with the same mean than the empirical sample were added: Blue, uniform distribution; Red, zero-truncated Poisson distribution; Green, power law degree distribution.

Fig 4 shows the empirical degree distribution of links between the different tiers in the seafood SC in the MM (vertical bars). The degree distributions for suppliers and retailers ($k_{s}^{out}$ and $k_{r}^{in}$) need to be taken with caution, since they were estimated mainly from imprecise information provided by wholesalers, not directly from suppliers and retailers.

As it can be observed in Fig 4, the in and out-distributions for wholesalers ($k_{w}^{in}$ and $k_{w}^{out}$) are quite homogeneous. In fact, a uniform or Poisson distributions can fit relatively well both distributions. A Poisson distribution roughly fits $k_{s}^{out}$ as well. However, the in-degree distribution for retailers $k_{r}^{in}$ is clearly heterogeneous, although the data size is extremely small to identify the specific degree distribution.

### Simulation setup

In order to test the influence of the network topology on the SC agility, we simulate the OFR performance in the SCRN model considering several degree distributions of firms in a tier. We limit the analysis to three stochastic distributions which represent the alternatives (homogeneous vs heterogeneous) we look for comparison.

Based on the empirical data above, we have considered a SC including 1000 firms distributed among the tiers according to the ratio 3:1:6, so we have 300 suppliers, 100 wholesalers and 600 retailers. The total demand from retailers follows the expression (1) with *θ* = 0.2. So, the total average capacity of wholesalers exceeds five times the demand at any time *t* excepting t*, when a shock occurs and the demand rises to the average capacity of wholesalers. The SCRN and the algorithm to calculate OFR was implemented in Matlab.

To simplify the results, we assume that every supplier trades with only one wholesaler, so the out-degree distribution $k_{s}^{out}$ is regular with mean degree equal 1. Additionally, we consider that wholesalers have the same behavior in their relations with suppliers and retailers, so the functional expression for wholesaler’s in- and out-degree distributions ${(k}_{w}^{in}\;\mathrm{and}\;k_{w}^{out})$ are the same. The probability functions are chosen in base of the empirical observation in section 4.1. Thus, three discrete degree distributions have been selected for $k_{w}^{in}\;\mathrm{and}\;k_{w}^{out}$ on the one hand, and for $k_{r}^{in}$ on the other hand:

- Uniform: The number of relationships between two firms adopts any value in a certain range with identical probability.
- Poisson: The number of relationships between two firms is close to a mean value.
- Power law: There are a short but significant number of firms accumulating many relationships, while the rest has few links.

The two first distributions are homogeneous, in the sense that no large capacity/demand differences among wholesalers/retailers are presented. On the contrary, the power law distribution is heterogeneous since it assumes a leading position of a firm with respect to another. Although the empirical data sample is too small to induce power law distribution, we use it in the simulations as a prototype of degree distribution generating a heterogeneous topology.

All distributions in the simulations are zero-truncated, so every node in the network has at least one link with a firm in the upper/lower tier. By means of this, we set aside the case of suppliers or retailers which are not integrated in the supply chain or wholesalers without relationships with either suppliers or retailers. In order to compare coherently the performance of the sampled supply networks, identical mean value is assigned for the three distributions. In particular, the mean in-degree of retailers is fixed at ${\bar{k}}_{r}^{in}=2$, which is the closest integer to the empirical mean degree 1.28 and higher than one.

We also assume two probability functions for the supplier capacity *c*_*s*_: a) Regular, where production is evenly shared among suppliers; b) Power law, where production is unevenly shared among suppliers.

### Results

**Fig 5:**
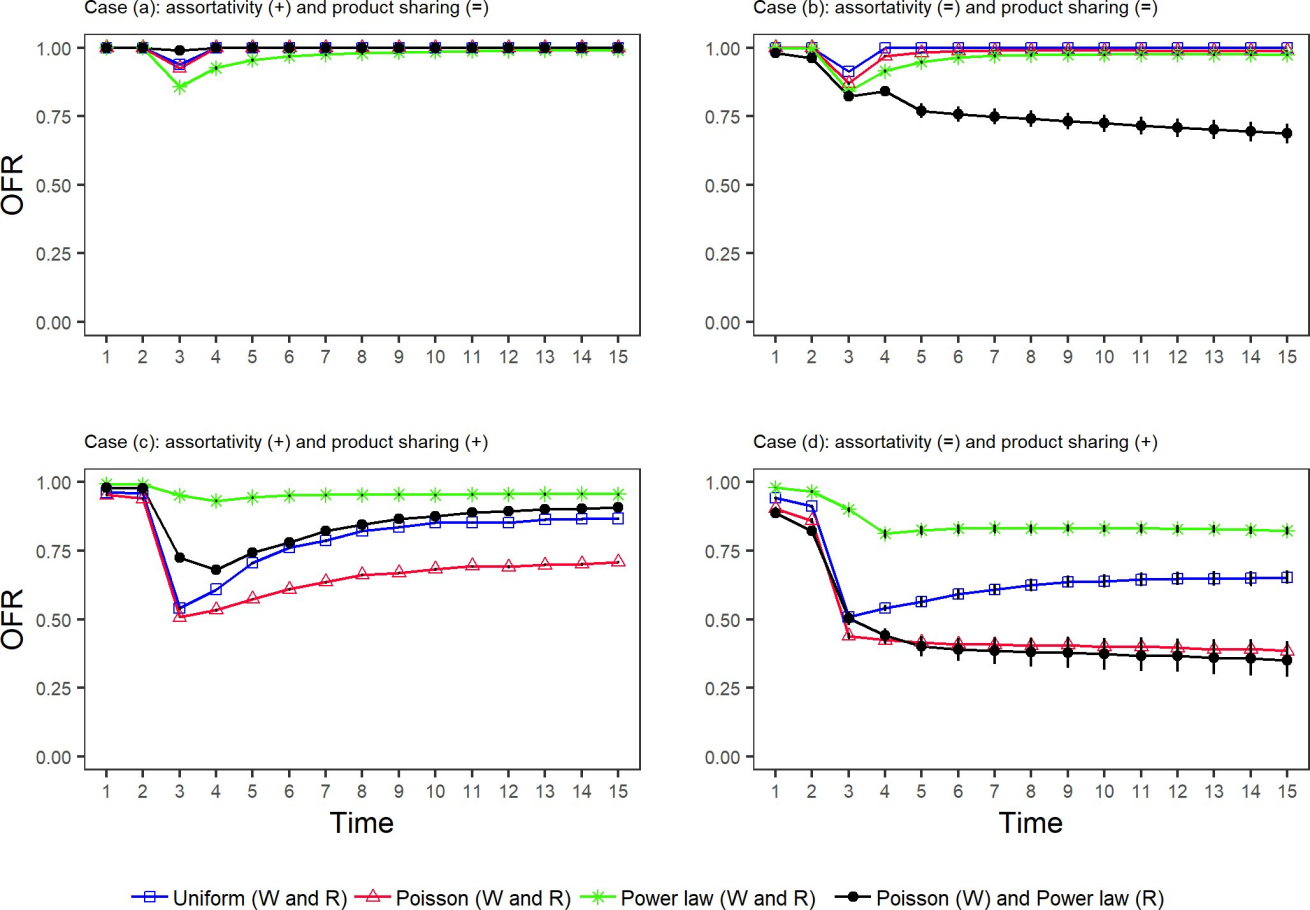
Order fulfillment rate (OFR) along 15 time steps for different wholesalers’ and retailers’ degree distributions. The retailers’ demand follows Eq (1) and demand shock occurs at t^*^ = 3. Four discrete distributions for the wholesalers’ (W) in- and out-degree ($k_{w}^{in}$ and $k_{w}^{out}$) and the retailers’ (R) in-degree ($k_{r}^{in}$) are assumed (blue square–Uniforms (W and R); red triangle–Poisson (W and R); green star—Power law (W and R); black bullet–Poisson (W) and Power law (R)). The model is simulated combining four different conditions: Assortativiy (+), where the relationships among wholesalers and retailers are ordered according to their degrees; Assortativity (=), where the relationships among wholesalers and retailers are randomly assigned; Product sharing (+), where the production is shared among suppliers by following a zero-truncated power law probability function; Product sharing (=), where the production is evenly shared among suppliers. The graph is obtained by doing 1000 simulations of the SCRN and taking mean values, samples were taken with a gap between the simulated and theoretical mean degree lower than 5%. The 95% confidence interval for the OFR mean values from simulation 800 to 1000 is also included as error bar. Technical details: (i) The ratio of the number of firms between tiers is 3:1:6, the number of wholesalers is *N*_*w*_ = 100 and there is not horizontal relationships; (ii) The mean in-degree of retailers is ${\bar{k}}_{r}^{in}=2$ and, to assure consistency, the mean degree ${\bar{k}}_{w}^{out}={\beta \bar{k}}_{r}^{in}={6\bar{k}}_{r}^{in}$ and ${\bar{k}}_{w}^{in}={\alpha \bar{k}}_{s}^{out}=3$; (iv) The sample of $k_{w}^{in}$ and $k_{w}^{out}$ is ordered, so the wholesaler with the highest in-degree has also the highest out-degree, and so on.

Fig 5 presents the OFR along 15 times steps for several combinations of the degree distributions included in the analysis. In every graph, three of the simulations assume that wholesalers and retailers follow the same degree distribution (blue square–Uniforms; red triangle–Poisson; green star—Power law). The fourth (black bullet trajectory) resembles the empirical observation in the MM by combining homogeneous and heterogeneous distribution of links. Specifically, it assumes that wholesalers’ degree follows a zero-truncated Poisson and retailers’ degree follows a zero-truncated power law. Table 1 presents the agility results from simulations in Fig 5. The figures are obtained by taking mean values of 1000 simulations of the SCRN. To test the robustness of the results, the 95% confidence interval of the OFR mean values from simulations 800 to 1000 is represented for every point in Fig 5 as an error bar. As it can be observed, the OFR values are fairly stable for this range of simulations. The complete simulation data for Fig 5 can be found in the supporting information (S1–S4 Files).

**Table 1:** Agility results for different wholesalers’ and retailers’ degree distributions. Immediate effect of the demand shock (Δ*OFR*(t*)) and recovery time (τ) for the simulations of cases a, b, c and d in Fig 5.

	assortativity (+) product sharing (=)		assortativity (=) product sharing (=)		assortativity (+) product sharing (+)		assortativity (=) product sharing (+)	
Degree distribution	Δ*OFR*(t*)	τ	Δ*OFR*(t*)	τ	Δ*OFR*(t*)	τ	Δ*OFR*(t*)	τ
**Uniform** **(W and R)**	-6.2%	1	-8.7%	1	-43.7%	>15	-44.3%	>15
**Poisson** **(W and R)**	-7.5%	1	-12.9%	1	-45.6%	>15	-48.9%	>15
**Power law** **(W and R)**	-14.2%	2	-15.6%	3	-4.0%	3	-6.7%	>15
**Poisson (W)** **Power law (R)**	-1.1%	1	-14.6%	>15	-25.9%	>15	-38.7%	>15

First, we analyze the three colored trajectories on cases (a) and (b), which assume that wholesalers and retailers follow an identical degree distribution and all suppliers have identical capacity. In Fig 5A, it is assumed that the retailer with the highest number of relationships trades with the wholesaler with the highest out-degree, and so on. This condition is also called positive assortativity. On the contrary, Fig 5B shows the OFR evolution assuming that relationships among wholesalers and retailers are independent on their respective degrees. As it can be observed, orders are completely fulfilled before the shock in demand in both cases (a) and (b). The shock provokes an immediate downturn in OFR for all distributions considered, although the gap is lower when assuming homogeneous degree distributions. Specifically, Table 1 shows that when the shock happens, OFR decreases 6.2% and 7.5% for Uniform and Poisson distributions, respectively, and 14.2% for Power law, when product is evenly shared among suppliers. In case of production is not evenly shared among suppliers, OFR also decreases less for Uniform and Poisson distributions than for Power law. The recovery time of the OFR is shorter for Uniform and Poisson distributions as well. Therefore, the SC agility is higher when degree distributions are homogeneous (Uniform or Poisson) than heterogeneous (Power law). Moreover, the simulation shows that positive assortativity enhances agility.

The results change markedly in case of assuming power law distribution among the suppliers’ capacity (cases (c) and (d) in Fig 5). When assuming positive assortativity between wholesalers and retailers, the topologies induced by the zero-truncated power law obtain the highest agility values (Fig 5C and sixth and seventh columns in Table 1). The shock in demand only reduces slightly the OFR (4%), which is recovered three time steps afterwards. This is not the case for SCs with homogeneous topologies (blue square and red triangle trajectories), for which the OFR suffers a sharp decline (between 43 and 46%), very slowly recovered in the subsequent steps but not completed before 15 time steps. When positive assortativity among wholesalers and retailers are not present (Fig 5D and Table 1, last two columns), the recovery is further slow.

Therefore, given the conditions of the SCRN, the results show that the most efficient degree distribution in terms of agility depends on how the product is shared across suppliers. Assuming that production is evenly shared, SCs with heterogeneous topologies are less agile than SCs with homogeneous topologies. These results confirm H1 above. However, when production is unevenly shared among suppliers, power law appears as a suitable degree distribution to assure high SC agility.

The simulation results for the topology that resembles the empirical sample are noteworthy (black bullet trajectories in Fig 1, last row in Table 1). This topology combines a homogeneous distribution of the wholesalers’ links and heterogeneous distribution of retailers’ links. This network structure fits the most agile SC of the four in case of considering identical capacity of suppliers and positive assortativity among wholesalers and retailers (Fig 5A and Table 1, second and third column). However, if positive assortativity is not present, agility heavily decreases. Agility further worsens when production is unevenly shared among suppliers (Table 1, last six columns).

We do not dispose of empirical information about which one of the four market conditions in Fig 5, represented by cases (a), (b), (c) and (d), fits the real SC. However, a priori the conditions for Fig 5C can be the closest to the real case for two reasons: First, it is plausible to assume that production is unevenly distributed among suppliers, since fish products are subject to environmental conditions and closed seasons which limit the product supply from some areas but not from others; Second, well positioned wholesalers are those able to manage the largest amount and variety of products and thereby they can attract high demanding retailers, what works in favor of positive assortativity.

**Fig 6:**
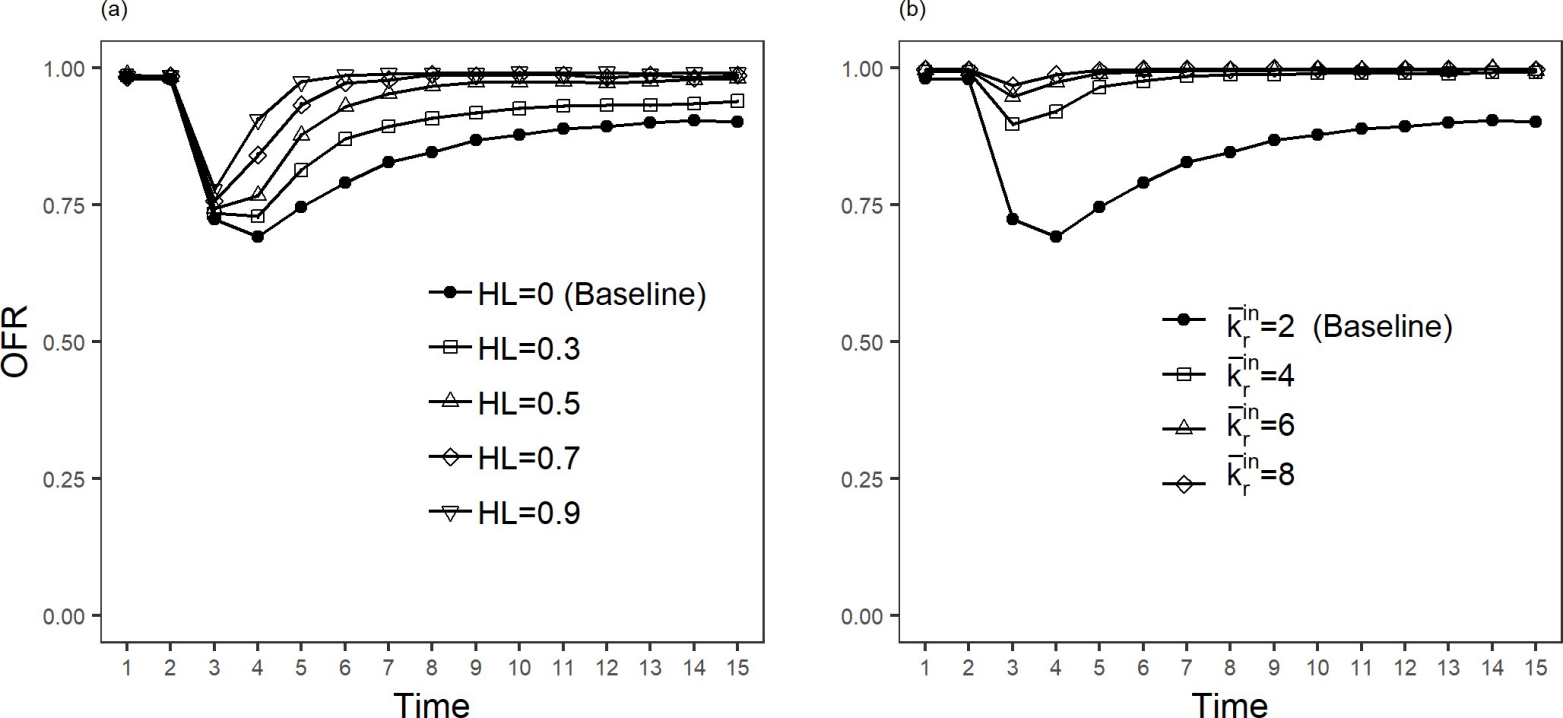
Order fulfillment rate (OFR) along 15 times steps for different percentage of horizontal links and mean in-degree of retailers. The baseline case corresponds to the conditions of Fig 5C: Wholesalers’ in- and out-degree ($k_{w}^{in}$ and $k_{w}^{out}$) are zero-truncated Poisson and retailers in-degree ($k_{r}^{in}$) is zero-truncated power law: (a) Results for five different percentages of wholesalers with horizontal links (HL); (b) Results for four mean in-degrees of retailers. The observed OFR is obtained by doing 1000 simulations of the SCRN and taking mean values.

The simulations above assume the absence of horizontal relationships in the SC. To validate H2, Fig 6A shows the OFR evolution when increasing the percentage of the total wholesalers with horizontal links (HL). The complete simulation data for Fig 6A can be found in S5 File. In the simulations we consider that two wholesalers with HL function as one, adding their capacities and orders. The baseline case (HL = 0) assumes the conditions for Fig 5C (black bullet trajectories). As it can be observed, the effect of the sudden change in demand is regularly dampened and the recovery time shortened when increasing the percentage of horizontal links. The agility measures for these cases are shown in the first three columns of the Table 2. Although the dampen effect on OFR is not heavy, the recovery time is substantially reduced starting from a percentage of horizontal links above 0.5. Half of interviewed wholesalers in the MM declared to have relationships with other wholesalers. The simulation results illustrate that this strategy has a positive and significant effect on the SC agility.

**Table 2:** Agility results for different percentage of horizontal links and mean in-degree of retailers. Immediate effect of the demand shock (Δ*OFR*(t*)) and recovery time (τ) for the simulations in Fig 6.

Horizontal links (HL)	Δ*OFR*(t*)	τ	mean in-degree of retailers	Δ*OFR*(t*)	τ
**HL = 0 (Baseline)**	-26.1%	>15	${\bar{k}}_{r}^{in}$ **= 2 (Baseline)**	-26.1%	>15
**HL = 0.3**	-25.2%	>15	${\bar{k}}_{r}^{in}$ **= 4**	-9.7%	2
**HL = 0.5**	-24.4%	4	${\bar{k}}_{r}^{in}$ **= 6**	-4.8%	1
**HL = 0.7**	-23.1%	3	${\bar{k}}_{r}^{in}$ **= 8**	-2.9%	1
**HL = 0.9**	-21.2%	2			

The effect of increasing vertical relationships on the SC agility is shown in Fig 6B (the complete simulation data can be found in S6 File). By raising the number of relationships among agents in different tiers, we analyze the scale effect on agility. Again, the conditions for Fig 5C (black bullet trajectories) were chosen as the baseline. The model was simulated assuming higher values of the mean in-degrees of retailers. To assure consistency, the mean out-degree of wholesalers and suppliers’ capacity increase consequently. The results in Fig 6B and last two columns in Table 2 show that both the sudden decrease of the OFR and the recovery time severely reduce when the mean number of relationships among firms increases.

These results confirm H2 and additionally inform that the marginal positive effect on agility of increasing the number of relationships is larger when starting from low interrelationships between wholesalers.

## Discussion and conclusions

This paper analyzes the influence of the pattern of relationships on the agility of a specific supply chain. SC agility means quick response to market changes and is one of the characteristics of an efficient SC. In particular, the paper analyzes the case of SCs where relationships are restricted to firms located in subsequent tiers. The restricted relationships between firms mean that each firm trades exclusively with partners located in the previous or next tier. This condition does not adjust to some industrial SCs, such as automotive (4), but does to FSCs including many small-scale producers located in rural areas and unable to take their products to outside markets [45–49]. In these circumstances, direct trading between retailers and producers is mostly absent, so intermediaries (middlemen and wholesalers) make a coordination role facilitating product transmission. This type of restrictions is also presented in military logistic networks.

The simulation model here is specifically designed for analyzing agility in SCs with restricted relationships. The algorithm includes the flow of product and order allocation rules. The results show that, when a shock in demand occurs, homogeneous distribution of links favors agility more than heterogeneous distributions of links if the product is evenly shared among suppliers. If this is not the case, SC structures where some few leader agents hold many trade relationships and the rest has few relationships are more agile than those with homogeneous distribution of links.

These findings are related to other previous theoretical and experimental contributions which analyze the influence of topology on SC resilience [31,33,34,41,55,63]. These contributions found that heterogeneous topologies characterize the most resilient SCs. The results in this paper illustrate that this is not the case for agility in SCs with restricted relationships. The restriction of product distribution across several echelons limits the efficiency of SCs with heterogeneous distribution of links among agents. In this kind of topologies, some intermediaries can suffer product shortage while others have excess when a demand shock occurs. This is not the case when all agents can commercialize each other directly, as it has been assumed in previous simulation models [33,41]. Our study also departs from these papers by analyzing the effect of demand shocks, instead of supply disruptions. Nevertheless, the results find that redundancy or increase in relationships enhances SC agility, in line with common recommendations given for resilient SCs [19,20].

The simulation results also show that SC agility increases with the presence of horizontal relationships between agents in the same tier. The beneficial effects of horizontal links was observed and tested in FSCs [22]. Our findings here agree and complete this and other previous theoretical and empirical analysis [18,64].

The agility metric proposed in this paper includes two factors: a) The immediate effect of sudden demand change on OFR; and b) the recovery time. Both factors give information about the quick response to the shock. Moreover, the influence of logistic flexibility on agility is implicitly estimated by comparing the results of different degree distributions. In terms of Swafford, Ghosh, and Murthy [16], the effect of range of states and timeliness are estimated by this metric. However, the cost factor is not captured. Other dimensions of agility are not measured by OFR as well, such as alertness or collaboration. In this paper, OFR is influenced exclusively by the structure of interrelationships among agents, not by any other quality of communication or alternative work design.

The topology of the sampled real FSC (the MM in Guadalajara, Mexico) combines homogeneous and heterogeneous distributions of links in the three tiers. The simulation results show that this structure is in between the most favorable one (heterogeneous distributions of links in all tiers) and other less favorable (homogeneous distributions of links in all tiers). Nevertheless, the existence of horizontal relationships among agents (up to 50%) increases SC agility significantly. Thus, from the simulation results followed in this paper, we conclude that the agility of the FSC in the MM is relatively high. Nevertheless, the results must be taken with caution, since information about suppliers and retailers in the sample is not complete.

In addition to the shortcomings of OFR as a metric for SC agility, we have to take into account the following limitations of the methodological approach: First, only demand changes are considered to estimate SC agility, given aside the effect of supply shocks; Second, inventory levels or delivery times are not assumed in the model; Third, the algorithm assumes a mechanistic way of trading, such as hierarchy in the order and supply allocation rules, while this rule is probably not applied by real agents, which may behave strategically; and Fourth, the conclusions are based on the outcome obtained with SC structures built from the combination of three specific degree distributions, so the optimal structure of a SC in terms of agility is not shown in this paper.

Having in mind these limitations, some managerial recommendations can be derived from our findings. They are circumscribed to those SCs in the conditions analyzed in the paper, i.e. restricted relationships among firms, where FSCs are a suitable example. High levels of agility are specifically desirable in these types of supply chains, since they manage perishable products that need fast distribution. Our results support the common belief on the goodness of heterogeneous topologies for SC efficiency, but only when this configuration is shared by all tiers. Thus, policies that allow the concentration of capacity in few agents (middlemen or wholesalers) are justified to enhance SC agility, but only in case suppliers’ capacity is also concentrated in few agents as well. Instead, low agility performance of the SC is expected if all suppliers have similar distribution capacity and wholesalers and retailers follow a heterogeneous topology. According to the results, promotion of cooperation among agents in the same tier (horizontal links) is also highly recommended.

This study can be extended in several ways. For example, this paper does not consider differences in the relationships among partners. However, strong and weak ties are present in SC relationships and show the degree of trade frequency or compromise between firms. The influence of the number and disposition of these ties on SC agility is still to be quantitatively analyzed.

## Supporting information

S1 File Simulation data corresponding to Fig 5A. (CSV)

S2 File Simulation data corresponding to Fig 5B. (CSV)

S3 File Simulation data corresponding to Fig 5C. (CSV)

S4 File Simulation data corresponding to Fig 5D. (CSV)

S5 File Simulation data corresponding to Fig 6A. (CSV)

S6 File Simulation data corresponding to Fig 6B. (CSV)
